# CUMS alters behavioral phenotypes and gut mycobiota in mice

**DOI:** 10.3389/fmicb.2026.1908564

**Published:** 2026-09-07

**Authors:** Yiyang Miao, Xinyuan Ma, Tong Feng, Xi Yi, Han Zhang

**Affiliations:** 1 College of Acupuncture and Tuina, Chengdu University of Traditional Chinese Medicine, Chengdu, China; 2 College of Health and Rehabilitation, Chengdu University of Traditional Chinese Medicine, Chengdu, China; 3 College of Medicine and Life Sciences, Chengdu University of Traditional Chinese Medicine, Chengdu, China

**Keywords:** *Aspergillus*, behavioristics, CUMS, fungi, gut mycobiota, ITS sequencing, *Kazachstania*, mice

## Abstract

**Objective:**

To investigate the effects of chronic unpredictable mild stress (CUMS) on the gut fungal community in mice and its association with anxiety-like behaviors.

**Methods:**

C57BL/6 male mice were randomly divided into a control group and a CUMS group, with 10 mice per group. The CUMS group was exposed to a 3-week regimen of chronic stressors (damp bedding, food and water deprivation, continuous light exposure, tail pinch, cage tilting, restraint, and forced swimming). Anxiety-like behaviors were assessed using the elevated plus maze (EPM) and open field test (OFT). Colonic contents were collected for ITS high-throughput sequencing. Alterations in the gut fungal community and their correlations with behavioral parameters were evaluated through α-diversity and β-diversity analyses, LEfSe differential analysis, Spearman correlation analysis and co-occurrence network analysis.

**Results:**

Compared with the control group, the CUMS group showed significantly reduced percentage of open-arm entries and percentage of time spent in open arms in the EPM (*P* < 0.01 and *P* < 0.001, respectively), as well as decreased central entries in the OFT (*P* < 0.05), indicating successful induction of anxiety-like behaviors. α-Diversity analysis revealed lower Chao and Shannon indices in the CUMS group (*P* < 0.05). β-Diversity analysis demonstrated a marked separation in fungal community structure between the two groups (*R* = 0.9420, *P* = 0.001). Species composition and LEfSe analysis identified *Kazachstania* as the absolutely dominant genus in the CUMS group (LDA score > 5.5), with its relative abundance being highly significantly increased (*P* < 0.001), while the relative abundances of most other dominant genera (e.g., *Aspergillus, Scopulariopsis*, and *Wallemia*) were significantly decreased. Correlation analysis revealed that the enrichment levels of *Aspergillus, Scopulariopsis*, and *Wallemia* were positively correlated with fungal species diversity (*P* < 0.05), and these taxa also exhibited positive correlations with the reduction in anxiety-like behaviors. Co-occurrence network analysis indicated that *Kazachstania* acted as the hub taxon with the highest connectivity within the network, exhibiting extensive niche antagonism against fungal taxa including *Aspergillus, Scopulariopsis*, and *Wallemia* in the community. In contrast, other fungal groups such as *Aspergillus, Scopulariopsis* and *Wallemia* showed pairwise positive co-occurrence relationships, forming a stable fungal subcommunity with internal synergistic interactions.

**Conclusion:**

This study firstly demonstrates that chronic unpredictable mild stress (CUMS) induces profound ecological remodeling of the gut fungal community, characterized by the enrichment of *Kazachstania* and suppression of other fungal genera. Combined with prior evidence, our results suggest CUMS enables *Kazachstania* to become conditionally dominant as an opportunistic fungus. We hypothesize CUMS-mediated homeostatic disturbance facilitates pathological overgrowth of *Kazachstania*, though this overgrowth does not directly exacerbate anxiety-like behaviors. By contrast, *Aspergillus, Scopulariopsis* and *Wallemia* may maintain intestinal homeostasis and alleviate anxiety-like phenotypes. These findings challenge the conventional view that fungal community dysbiosis is merely reflected by reduced diversity; instead, monotypic fungal dominance better mirrors pathological status. This work provides novel theoretical perspectives and research directions for exploring how CUMS shapes host health via gut mycobiota.

## 1 Introduction

Anxiety disorders are mental health problems that result from a combination of different causes and are mainly characterized by low mood and helplessness (Li et al., 2024). In severe cases, self-injurious behavior or suicidal behavior may develop. Disorders of this kind frequently have a chronic course, are unresponsive to treatment, and exhibit high recurrence risk, with considerable impairment in daily life. The World Health Organization (WHO) data reveals that by 2025, one billion people will be suffering from mental disorders. The most common of all ailments will be anxiety and depression. The WHO estimates a loss of US $1 trillion due to anxiety and depression. According to the 2019 China Mental Health Survey, the lifetime prevalence of any anxiety disorder among Chinese adults is 7.6% and that of any depressive disorder is 6.8%. Furthermore, major depressive disorder (MDD) with a lifetime prevalence of 3.4% occurs across the lifespan. There is sufficient documentary evidence to show that anxiety disorders have become one of the leading global health burdens in the world. It is because of the high rates of suicide due to anxiety disorder that the suffering of the individual has an enormous clinical and economic cost to society. Standard clinical management of anxiety disorders involves stage-dependent prescription of antidepressants, including tricyclic antidepressants and selective serotonin reuptake inhibitors (SSRIs) (Li et al., 2024). However, most clinically used anxiolytics elicit adverse reactions to varying degrees, and due to individual differences, some patients show insufficient response. Given the high prevalence of anxiety disorders, the search for novel and broadly applicable therapeutic strategies remains a primary focus of anxiety disorders research.

The gut microbiota is a consortium of microorganisms encompassing bacteria, fungi, viruses, parasites, and archaea. Through bidirectional communication pathways between gut microbes and the host's central nervous system, it interacts with mental disorders such as anxiety disorders (Ma et al., 2023). To date, research on the relationship between the gut microbiota and human pathology has largely focused on the bacterial microbiome. Nevertheless, recent evidence indicates that over 400 species of gut mycobiota are associated with humans and play significant roles in disease onset and progression. Dysbiosis of the gut mycobiota directly disrupts microbial community balance, thereby affecting the growth of pathogenic microorganisms and interspecies competition (Zhang et al., 2022a). It interacts with the host transcriptome, epigenome, metabolome, and immune system to cause disease through diverse mechanisms and outcomes (Zhang et al., 2022b). The internal transcribed spacer (ITS) is a non-coding intergenic region situated between the 18S, 5.8S, and 28S rRNA gene clusters in the fungus. It shows a high degree of interspecies variability, along with conserved flanking regions and multiple copies. This makes it ideal for the development of universal primers used in amplification and detection. This facilitates differentiation and identification of various fungal species. The potential of high-throughput sequencing tools such as ITS to reveal the compositional differences in gut fungi of patients and healthy controls may further our understanding of disease pathogenesis, clarify disease progression and outcomes, and ultimately lead to the development of drugs and their treatment in the clinic.

Society is highly affected by anxiety disorders. The CUMS model promotes anxiety or depression under stressful conditions and is the most widely used model to study the mechanisms of these two under chronic psychological stress (White et al., 2024). Notably, the underlying pathogenesis remains not fully understood. Furthermore, the existing studies do not investigate the association between anxiety disorders and the gut mycobiota. The objective of this study is to evaluate if and how the gut fungal community changes in CUMS mice and which way these changes happened. Moreover, we will conduct behavioral tests of mice to verify the link between these changes and the CUMS model, which will set the stage for future mechanistic analyses as well as clinical applications.

## 2 Materials and methods

### 2.1 Animals and grouping

In this experiment, we used specific pathogen-free (SPF) grade male C57BL/6 mice. Each mouse aged 7 weeks with an average body weight of 20 ± 2 g, was housed under a 12-h light/dark cycle, except during certain modeling procedures. After a 1-week adaptation period, we randomly assigned the mice to a control group (Con) or a chronic unpredictable mild stress (CUMS) group (Mod), with 10 mice per group.

This sample size satisfies the statistical requirements for behavioral tests and gut mycobiota sequencing analyses (Zhang et al., 2021). Cyclic fluctuations of sex hormones during the estrous cycle in female mice would cause unstable emotional and behavioral performance, accompanied by spontaneous variation in intestinal microbial composition. Male mice were selected to eliminate this confounding factor and stabilize the data obtained from behavioral tests and gut fungal sequencing (Liu et al., 2021). The CUMS group was exposed to a 3-week CUMS protocol. The control group was maintained under standard housing conditions throughout the same period.

All experimental protocols complied with the National Institutes of Health (NIH). *Guidelines for the Care and Use of Laboratory Animals* and were approved by the Institutional Review Board.

### 2.2 Preparation of the CUMS model

Without exposure to any stressors, the control group was kept under standard environmental conditions. The CUMS group was subjected to the following stressors in a randomized sequence each week, with no identical stressor applied on two consecutive days: damp bedding (24 h), continuous illumination (24 h), food and water deprivation (24 h), tilted cage (24 h), physical restraint (1 h), tail pinch stimulation (2 min) and forced swimming (5 min). After 3 weeks, we evaluated the efficacy of stress induction via the elevated plus maze (EPM) and open field test (OFT; Tables 1, 2).

**Table 1:** Operational details.

No.	Stressor name	Duration	Operation (All other breeding conditions remain unchanged)
1	Damp bedding	24 h	Evenly soak all bedding in the cage with approximately 500 mL purified water
2	Continuous illumination	24 h	Expose cages to constant light conditions
3	Food and water deprivation	24 h	Remove animal feed and drinking bottles from cages
4	Tilted cage	24 h	Place the cage at a 45° inclined angle
5	Physical restraint	1 h	Seal mice into self-made ventilated restraint tubes to restrict free movement
6	Tail pinch stimulation	2 min	Clamp the middle segment of the mouse tail with hemostatic forceps; the stimulus induces painful sensation without causing severe tissue injury
7	Forced swimming	5 min	Prepare purified water at 20 °C with a water depth higher than the vertical standing height of mice hind limbs. Force mice to swim; manually intervene to keep mice swimming once they show floating and resting behavior

**Table 2:** The schedule of CUMS stressors.

Week	Days						
	Mon.	Tue.	Wed.	Thur.	Fri.	Sat.	Sun.
Week 1	7	5	3	1	2	4	6
Week 2	3	4	7	6	1	2	5
Week 3	1	6	2	5	4	3	7

### 2.3 Behavioral testing

Each mouse was put in the maze for a 5-min acclimatization period in the EPM test double block to get acquainted with the maze. The mouse was placed in the center, and over the 5 min, the frequency of entries into the open arms (open-arm entries, OE) and the duration of stay in the open arms (open-arm time, OT) were monitored. After the trial, we computed the percentage of entries into the open arms (OE%) as well as percentage of time spent in the open arms (OT%) as follows. OE% = [open-arm entries/(open-arm entries + closed-arm entries)] × 100%. The percentage of time spent in the open arms is calculated as: OT% = [open-arm time/(open-arm time + closed-arm time)] × 100%.

Prior to the initiation of the open field test (OFT), individual mice were gently placed into the open field apparatus and allowed to acclimatize to the environment for 10 mins. The mouse was then quickly placed in the central area. The operator left the apparatus and recorded, over 5 min, the number of crossings of the outer perimeter squares, the number of crossings of the central square, and the number of rearing episodes (crossing a square was defined as all four paws entering the square; rearing was defined as both forelimbs raised at least 1 cm above the floor).

After each mouse completed the test, the apparatus was disinfected with 75% ethanol to prevent residual odors from affecting the subsequent mouse.

Prior to performing statistical analysis, one maximum and one minimum value were symmetrically removed from the behavioral data of each group (*n* = 8). Extreme outliers have the potential to inflate pooled variance within groups, and obscure true behavioral differences between groups. The application of symmetric trimming effectively reduces this bias, thereby increasing statistical power (Vankov, 2023; Wilcox, 1995; Mehrhoff et al., 2023).

### 2.4 Sample collection and preparation

Following isoflurane anesthesia, all mice were decapitated. Colonic contents were collected under sterile conditions, immediately frozen in liquid nitrogen, and stored at −80 °C after completion of all sampling procedures.

### 2.5 Gut microbiome analysis

Gut fungal analysis was conducted by Shanghai Meiji Biotechnology Co., Ltd., Shanghai, China. DNA was extracted from colonic content samples, and the extracted genomic DNA was examined by 1% agarose gel electrophoresis. Specific barcoded primers targeting the designated sequencing regions were synthesized and used for PCR amplification. All samples were processed under standard experimental conditions in triplicate. The PCR products which originated from the same sample were pooled and run on 2% agarose gels. Subsequently, the AxyPrep DNA Gel Recovery Kit was utilized to purify the products. They were subjected to elution with Tris-HCl, followed by analysis through 2% agarose gel electrophoresis. Based on preliminary quantification by electrophoresis, PCR products were quantified using the QuantiFluor^TM − ST^ Blue Fluorescence Quantification System (Promega). Amplified region: ITS1 fragment.

Primers: ITS1F (5′-CTTGGTCATTTAGAGGAAGTAA-3′) and reverse primer ITS2R (5′-GCTGCGTTCTTCATCGATGC-3′). Sequencing platform: Illumina NextSeq 2000. Taxonomic annotation database: UNITE 9.0. Taxonomic classification algorithm: Classify-sklearn (Naive Bayes classifier), classification confidence threshold set at 0.7. Sequencing depth threshold: 50,000 clean reads per sample.

To minimize the confounding effects of uneven sequencing depth on subsequent alpha and beta diversity analyses, all samples were rarefied to an equal sequencing depth of 40,583 reads. Following rarefaction, the average Good's coverage across all samples remained exceptionally high at 99.9%. Samples were mixed in appropriate proportions according to sequencing requirements to construct the library. Sequencing was performed on the instrument, and the fluorescence signal data collected from each PCR cycle were analyzed to determine the sequence of the template DNA fragments.

### 2.6 Statistical analysis

**Figure 1:**
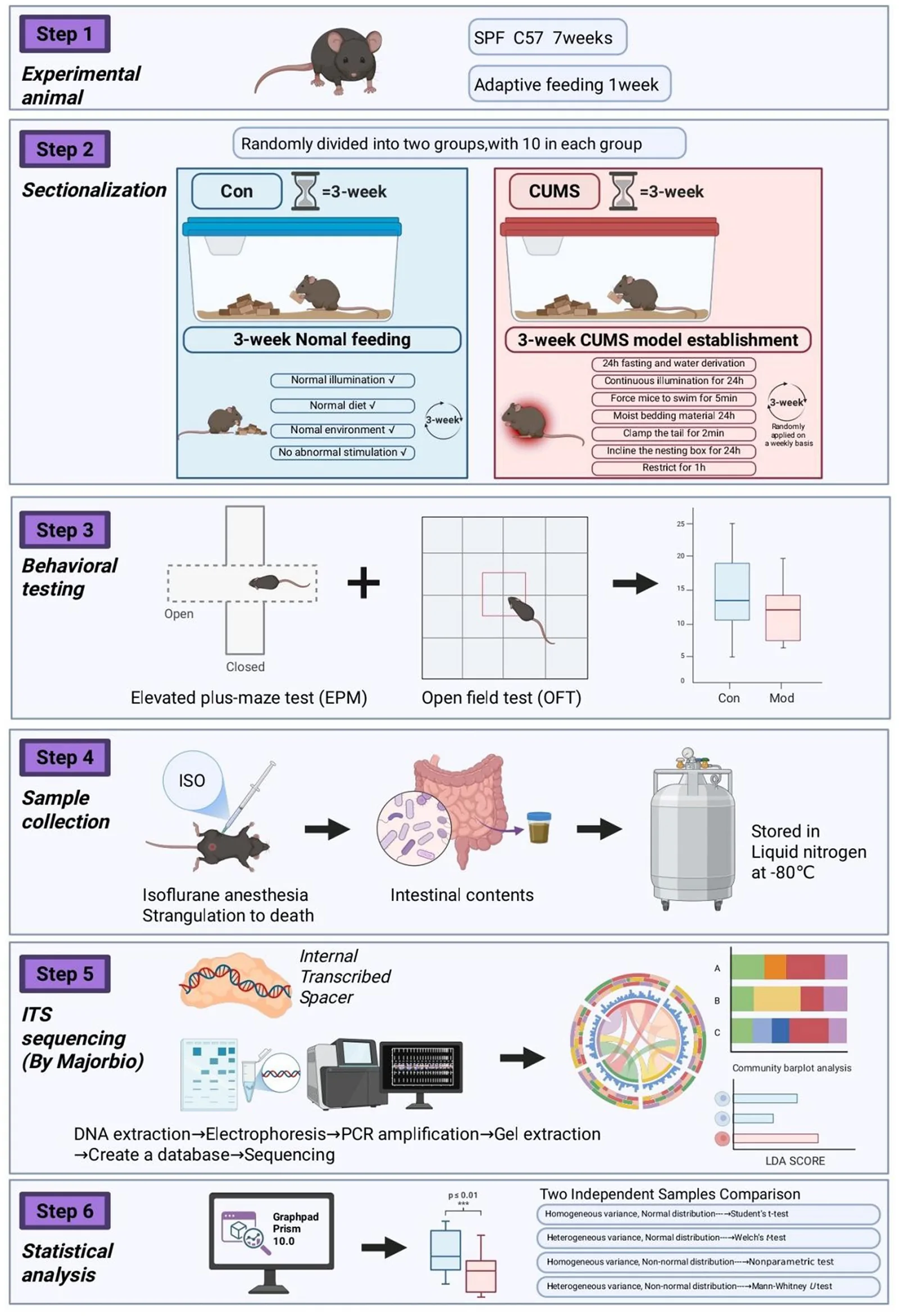
Flowchart of the ITS sequencing experiment for gut fungi in the CUMS mouse model of depression. This figure illustrates the experimental procedure for ITS sequencing of gut fungi in CUMS-induced depressed mice: after acclimatization, mice were assigned to groups and subjected to modeling; following behavioral validation, intestinal samples were collected; fungal communities were analyzed by ITS sequencing; and finally, statistical analyses were performed.

Following sample splitting based on sequencing results, quality control and sequence assembly were performed. The data were processed and optimized using sequence denoising methods to obtain amplicon sequence variant (ASV) representative sequences and abundance information for downstream analyses. All data were analyzed and plotted using GraphPad Prism 10.0 software. Quantitative data were expressed as mean ± standard deviation (SD). For pairwise comparisons, Student's *t*-test was used when variances were homogeneous, and Welch's *t*-test was used when variances were heterogeneous. A *P*-value <0.05 was considered statistically significant (Figure 1).

## 3 Results

### 3.1 Ethology

#### 3.1.1 Elevated plus maze (EPM) test

To evaluate anxiety, we assessed anxiety-like behavior using the elevated plus-maze (EPM) paradigm. The primary outcome measures included the percentage of open arm entries (OE%), the percentage of time spent in the open arms (OT%), the total time spent in the open arms, and the number of entries into the open arms. The maximal and minimal data points were excluded from each group (original *n* = 10), leaving *n* = 8 biological samples for statistical comparison. The results are presented as follows.

**Figure 2:**
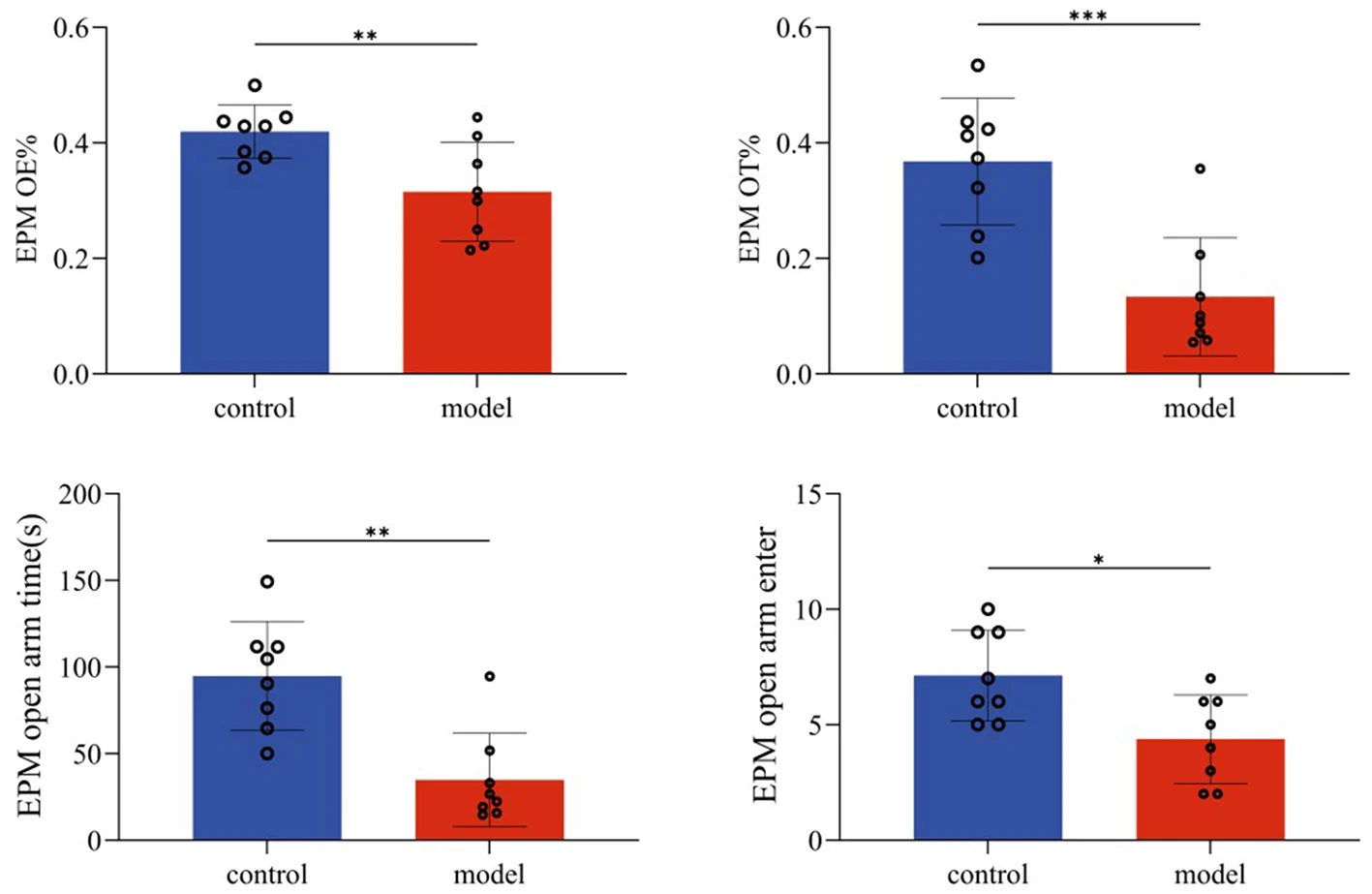
Behavioral index analysis of the elevated plus maze in the Con group and CUMS group (regular bar chart and statistical estimation plot). Con represents the Con group, Mod represents the CUMS group, *n* = 8, * indicates *p* < 0.05, ** indicates *p* < 0.01, and *** indicates *p* < 0.001.

**Figure 3:**
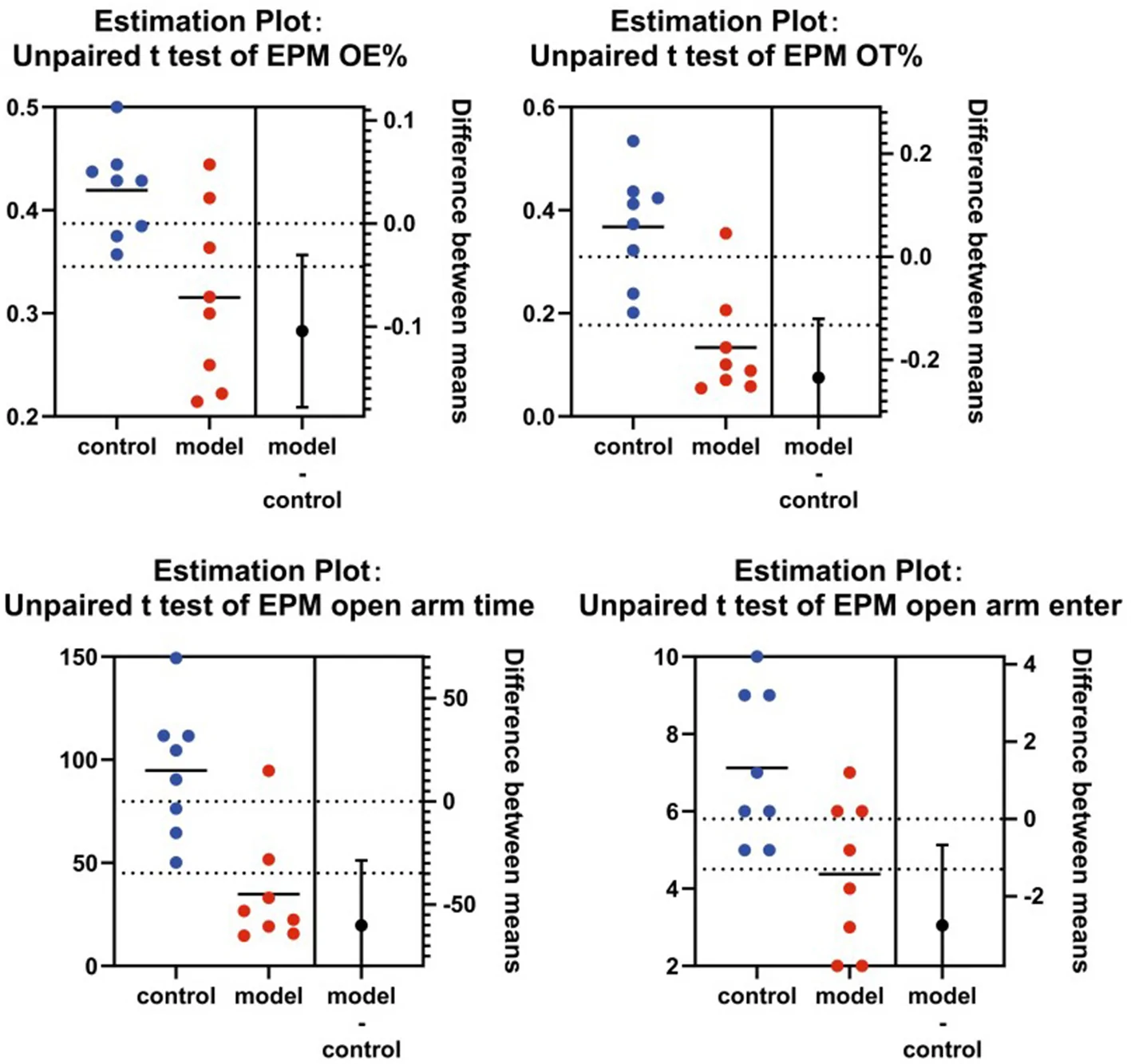
Behavioral index analysis of the elevated plus maze in the Con group and CUMS group (regular bar chart and statistical estimation plot). Con represents the Con group, Mod represents the CUMS group, *n* = 8, * indicates *p* < 0.05, ** indicates *p* < 0.01, and *** indicates *p* < 0.001.

Figures 2,3 present the standard bar charts and statistical estimation plots for core EPM indicators in both the Control (Con) and Chronic Unpredictable Mild Stress (CUMS) groups. As compared to the Con group, the CUMS group revealed a significantly lower percentage of open entries (OE%) and an extremely significant lower percentage of time in the open arms (OT%) and also a significantly lower time spent in the open arms with fewer open arm entries. It was confirmed that these differences were statistically significant according to estimation plots and statistical analyses. The CUMS group displayed differences in inhibitory tendencies for exploratory activity in the elevated plus-maze, sampling less in the open arms and spending less time in the open arms than the Con group, according to the analysis.

#### 3.1.2 Open field test

The open field test is utilized to assess anxiety-like behaviors and spontaneous locomotor activity in mice. Key observation indicators include the number of entries into the central zone, the duration of stay in the central zone, and the total distance traveled. The maximal and minimal data points were excluded from each group (original *n* = 10), leaving *n* = 8 biological samples for statistical comparison. The results are presented below.

**Figure 4:**
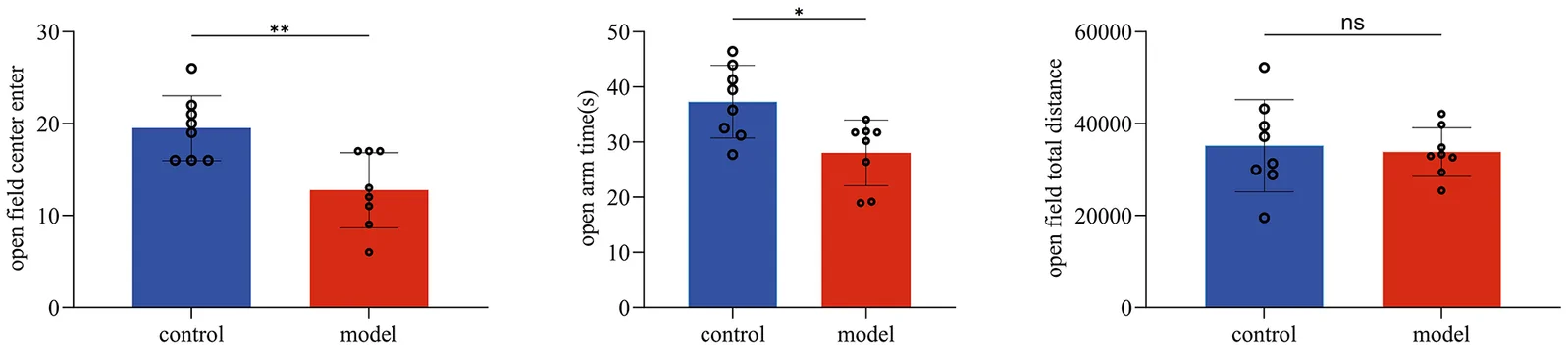
Behavioral index analysis of the open field test between the Con group and the CUMS group (regular bar chart and statistical estimation plot). Con represents the Con group, Mod represents the CUMS group, *n* = 8, * indicates *p* < 0.05, ** indicates *p* < 0.01, ns indicates no statistical significance.

**Figure 5:**
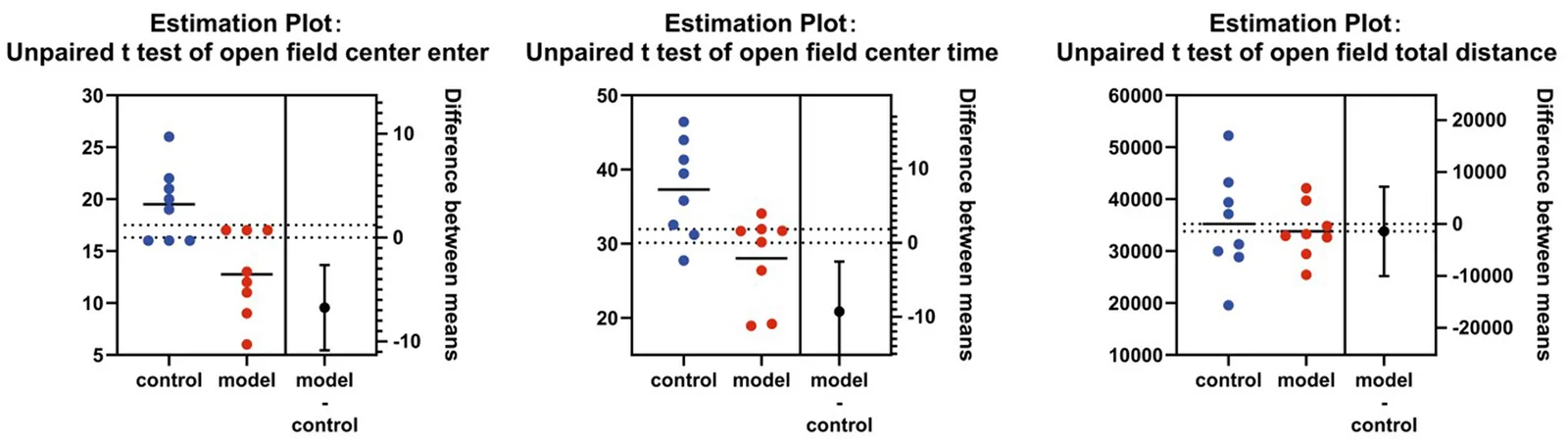
Behavioral index analysis of the open field test between the Con group and the CUMS group (regular bar chart and statistical estimation plot). Con represents the Con group, Mod represents the CUMS group, *n* = 8, * indicates *p* < 0.05, ** indicates *p* < 0.01, ns indicates no statistical significance.

The general bar graphs and statistical estimation plots of core indicators in the open field test of each group of mice are shown in Figures 4, 5, respectively. The chronic unpredictable mild stress (CUMS) group (Mod) had a statistically significant decrease in the number of entries into the central grid and the time spent in the central grid compared to the control group (Con). But the total distance traveled changed very little and was not significant. After CUMS modeling, the mice explored the central area of the open field less, although their total locomotor activity did not change significantly. Furthermore, it was noted that the CUMS group spent significantly longer in the peripheral area of the open field and showed more wall-hugging behavior. Changes in behavior can signify the onset of anxiety problems.

### 3.2 Gut mycobiota

#### 3.2.1 Alpha diversity

Amplicon Sequence Variants (ASVs) represent a method for the precise partitioning and identification of sequences generated by high-throughput sequencing. This approach facilitates the detection of biologically relevant, unique sequences present in various samples. Compared to traditional Operational Taxonomic Unit (OTU) methods, ASVs provide enhanced resolution and improved reproducibility.

**Figure 6:**
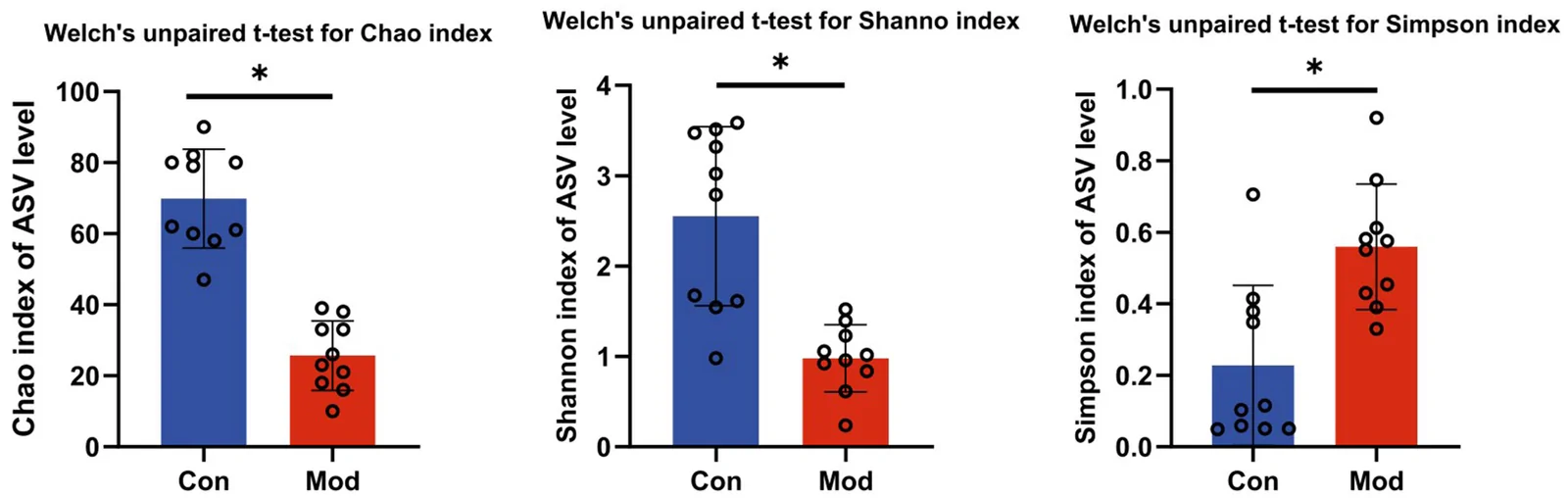
Analysis of α-diversity indices at ASV level for fungal communities between the Con group and the CUMS group. Con represents control group, Mod represents CUMS group, *n* = 10; * indicates *P* < 0.05, analyzed by Welch's unpaired *t*-test; A is Chao index, B is Shannon index, and C is Simpson index.

At the ASV level, Welch's unpaired *t*-test was performed to compare the α-diversity of fungal communities in the Control group (Con) against those in the Chronic Unpredictable Mild Stress group (Mod). The analysis included the Chao and Shannon and Simpson indices, which measure microbial species abundance and evenness. As illustrated in Figure 6, the CUMS group had decreased Chao and Shannon indices but an increased Simpson index compared to the Con group (*P* < 0.05). The species richness of gut mycobiota can be assessed by the Chao index. The Shannon and Simpson indices, however, reflect its diversity, meaning a higher Shannon index with a lower Simpson index corresponds to greater diversity. Conditions in which chronic unpredicted mild stress (CUMS) appears can reduce the Chao and Shannon indices and raise the Simpson index compared to control. The findings indicate that after modeling CUMS, the mice had a decline in gut mycobiota diversity and abundance but an increase in evenness.

#### 3.2.2 Beta diversity

**Figure 7:**
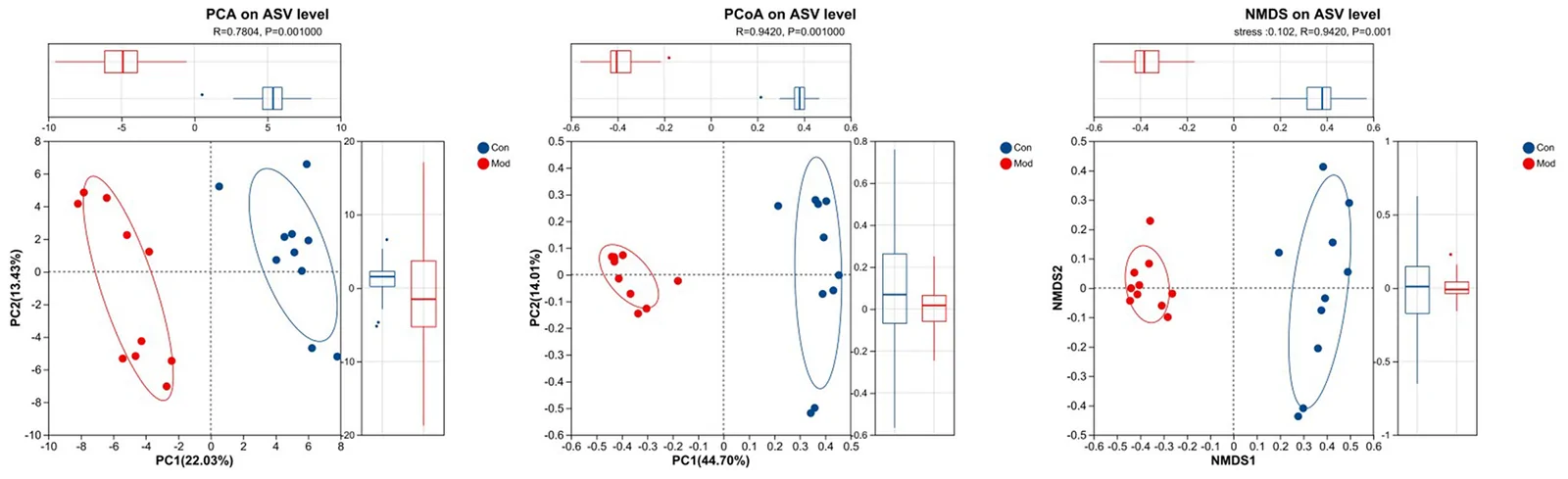
β-diversity analysis of fungal communities at ASV level between the Con group and CUMS group. Con represents Con group, Mod represents CUMS group, *n* = 10; A is a PCA plot with PC1 explaining 22.03% of variance; B is a PCoA plot with PC1 explaining 44.70% of variance; C is a NMDS plot with stress = 0.102, *R* = 0.9420, and *P* = 0.001.

Beta diversity analysis serves as a valuable tool for evaluating changes in the structure of gut mycobiota across different groups. We performed beta diversity analysis of fungal communities between the Control group (Con) and the Chronic Unpredictable Mild Stress (CUMS) group (Mod) at the Amplicon Sequence Variant (ASV) level, employing Principal Component Analysis (PCA), Principal Coordinates Analysis (PCoA), and Non-metric Multidimensional Scaling (NMDS). The results (Figure 7) indicated that in the PCA, the first principal component (PC1) explained 22.03% of the variance, while in the PCoA, PC1 accounted for 44.70% of the variance. Both analyses exhibited a clear trend of separation between the two sample groups along the coordinate axes. The NMDS analysis produced a stress value of 0.102 (<0.2), indicating high reliability of the results. Furthermore, this analysis demonstrated *R* = 0.9420 and *P* = 0.001, confirming an extremely significant difference (*P* < 0.001) in fungal community structure between the Con and CUMS groups. Collectively, these findings indicate that CUMS modeling significantly altered the overall structure of fungal communities, resulting in pronounced compositional divergence between the Con and the CUMS groups, with inter-group differences substantially exceeding intra-group variations.

#### 3.2.3 Analysis of microbial community composition at the phylum level

**Figure 8:**
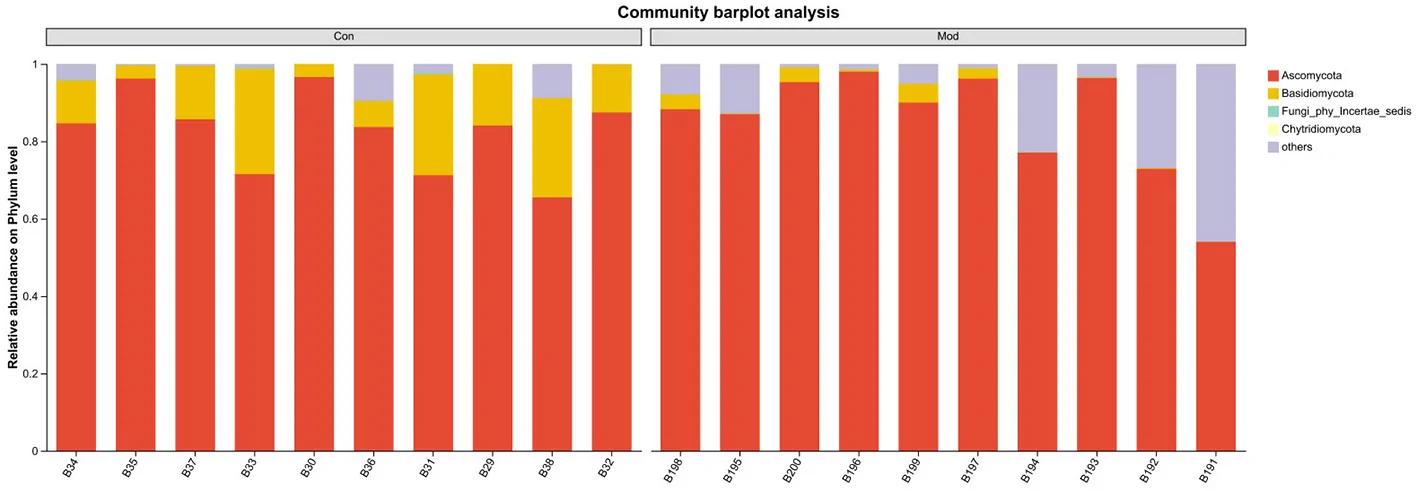
Relative abundance analysis of fungal community species composition between the Con group and the CUMS group. Con represents the Con group, Mod represents the CUMS group, *n* = 10; this figure shows the phylum-level community composition bar chart; “Others” refers to the sum of low-abundance taxa not listed in the figure.

Figure 8 illustrates the composition analysis of mycobiota at the phylum level, along with the relative abundance of species within these phyla. Compared to the control group (Con), the community composition of the chronic unpredictable mild stress (CUMS) group (Mod) exhibited no significant changes at the phylum level. In both groups, the fungal communities were predominantly dominated by *Ascomycota*, which served as the absolute dominant taxon. Visually, *Basidiomycota* appeared more abundant in the control group and less prevalent in the CUMS group.

#### 3.2.4 Analysis of microbial community composition at the genus level

**Figure 9:**
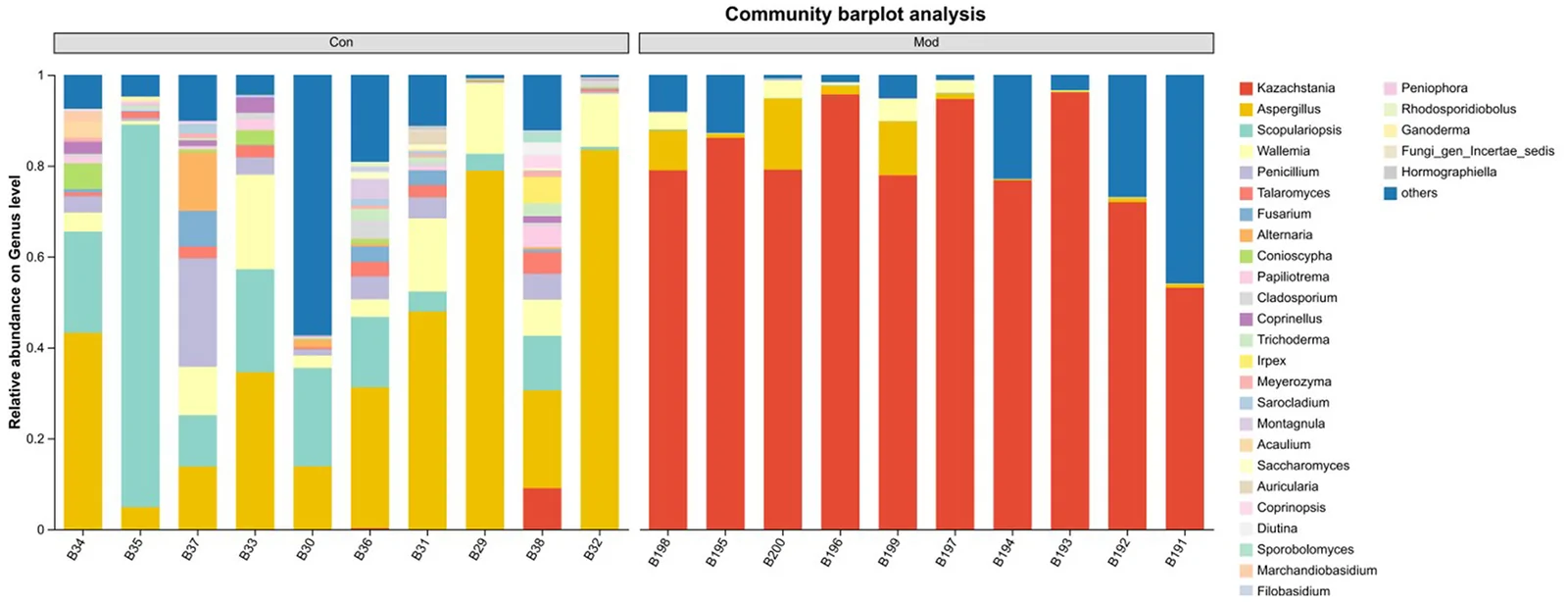
Comparison of differential species abundance at the genus level (ASV level) between the Control group and CUMS group in fungal communities. Con represents the Con group, Mod represents the CUMS group, *n* = 10; the horizontal axis shows the relative abundance percentage of species and the intergroup abundance difference percentage, respectively, while the vertical axis indicates the fungal genera with significant differences.

Statistical analysis of the differences in fungal community abundance at the genus level between the Control group (Con) and the Chronic Unpredictable Mild Stress (CUMS) group revealed (Figure 9) that significant changes occurred at the genus level, in contrast to the phylum level. The relative abundance of *Kazachstania* in the CUMS group was markedly higher than that in the Con group, indicating a significant positive increase in intergroup abundance differences. Conversely, dominant genera such as *Aspergillus, Scopulariopsis, Wallemia*, and *Penicillium* exhibited greater relative abundance in the Con group, all showing significantly negative decreases in intergroup abundance differences. Compared to the Con group, the CUMS group displayed a more simplified species composition with fewer species, where *Kazachstania* was predominant, and the abundance of other fungal genera was significantly lower than that of *Kazachstania*.

#### 3.2.5 Species difference analysis

**Figure 10:**
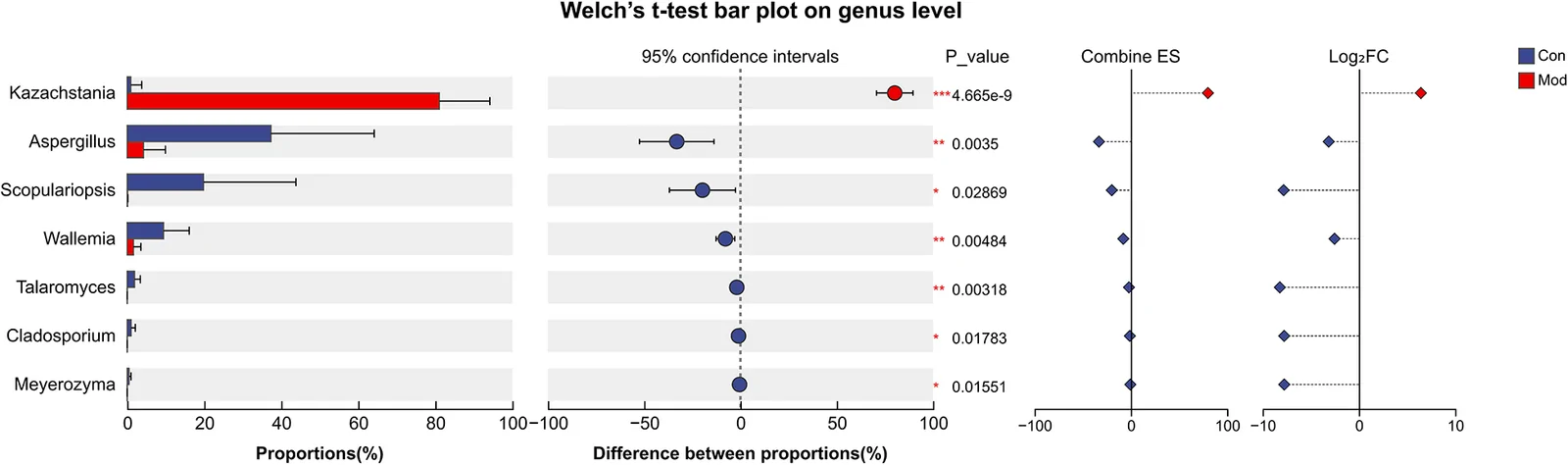
Welch's *t*-test analysis of differential fungal taxa at the genus level (ASV level) between the Con group and the CUMS group. Con represents the Con group, Mod represents the CUMS group, *n* = 10; the vertical axis indicates differential fungal genera, and the horizontal axis represents species abundance-related statistics.

At the level of amplicon sequence variants (ASVs), a Welch's *t*-test was performed to assess differences in fungal communities at the genus level between the Con group and the CUMS group. Seven distinct fungal genera exhibited significantly varied abundances between the two groups (*P* < 0.01, Figure 10). Notably, *Kazachstania* displayed an extremely significant difference between the two groups (^***^P < 0.001), while *Aspergillus, Wallemia*, and *Talaromyces* presented significant distinctions (^**^P < 0.01). *Scopulariopsis, Cladosporium*, and *Meyerozyma* showed only marginally significant differences (^*^P < 0.05). In terms of relative abundance variations, *Kazachstania* emerged as the predominant differential genus in the CUMS group, exhibiting a relative abundance considerably higher than that found in the Con group. Conversely, the relative abundances of the other six differential genera were significantly elevated in the Con group when compared to the CUMS group. These findings indicate that CUMS modeling has a species-specific impact on the abundance of various fungal genera, particularly reducing the prevalence of most dominant fungal genera while facilitating the growth of *Kazachstania*.

For the LEfSe analysis, a tailored LDA cutoff of 5 was utilized, focusing solely on taxa that had LDA scores surpassing this limit. The bar chart illustrates the true LDA effect sizes for each taxon. It is important to note that several taxa reached peak scores nearing 5.5, which indicates the results observed rather than the predefined screening threshold; consequently, a consistent filtering criterion was established at 5 (Segata et al., 2011; Zhang et al., 2023).

In the Con group, the enriched signature fungi primarily comprised three major branches. First, the *Eurotiales* order under the *Eurotiomycetes* class, along with the *Aspergillaceae* family and *Aspergillus* genus, exhibited the highest LDA scores in the Con group (approximately 5.5), representing the core features of this group. Second, the *Microascales* order under the *Sordariomycetes* class, together with the *Microascaceae* family and *Scopulariopsis* genus, had LDA scores exceeding 5, although slightly lower than those of the first branch. Lastly, the *Basidiomycota* phylum and its subordinate *Wallemia* order both recorded LDA scores above 4.5.

In the CUMS group, the enriched signature fungi were relatively homogeneous, consisting of *Saccharomycetes* (class), *Saccharomycetales* (order), *Saccharomycetaceae* (family), and *Kazachstania* (genus) under the *Ascomycota* phylum. Among these five taxa, all except *Ascomycota* exhibited LDA scores greater than 5.5, representing the most distinctive characteristics of this group.

In summary, the Con group was characterized by differential species such as *Aspergillu*s, *Scopulariopsis*, and the *Wallemia* order of *Basidiomycota*, while the CUMS group was marked by the presence of *Kazachstania*.

### 3.3 Correlation analysis

#### 3.3.1 Result description

**Figure 11:**
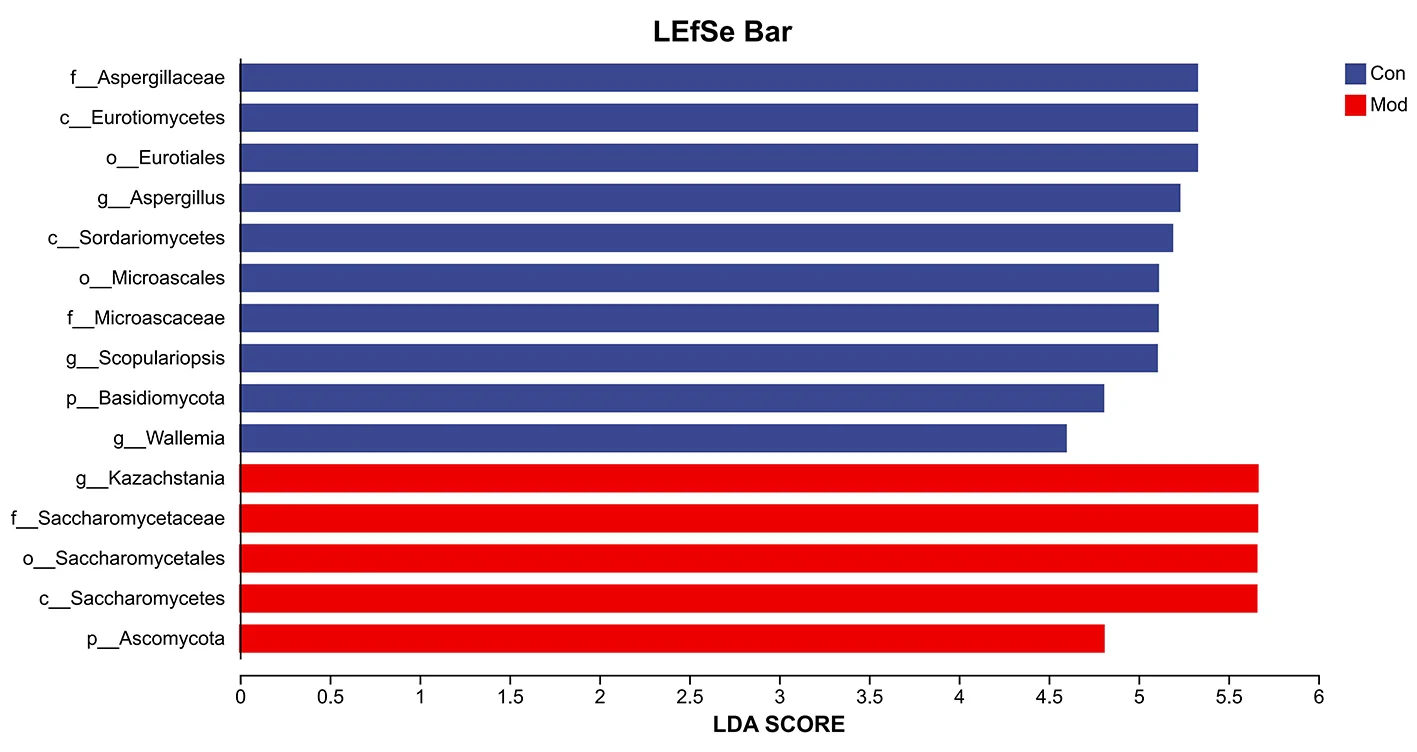
LEfSe analysis for biomarker screening of fungal community ASVs between the Con group and the CUMS group. Con represents the Con group, Mod represents the CUMS group, *n* = 10; the horizontal axis shows LDA scores, the vertical axis displays fungal taxonomic groups with statistically significant differences; different colors indicate group-specific biomarkers.

**Figure 12:**
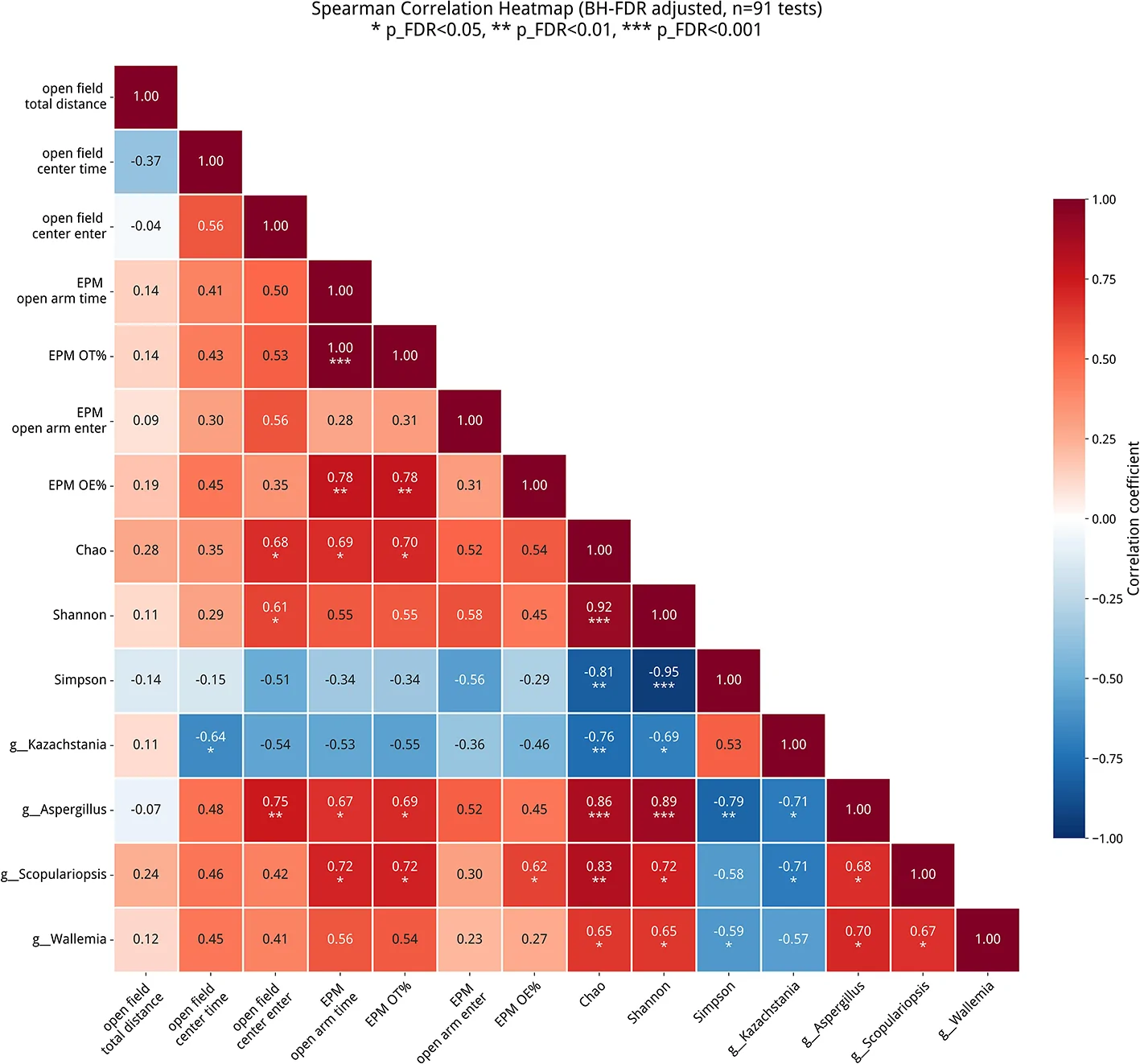
Heatmap of correlations between gut fungal diversity, key fungal genera and anxiety-like behaviors in mice. Spearman correlation analysis was employed, with red indicating positive correlations and blue indicating negative correlations. In this correlation analysis, we employed the FDR correction method. The color intensity is proportional to the absolute value of the correlation coefficient. Asterisks denote significance levels: *P < 0.05, **P < 0.01, and ***P < 0.001. Simpson, Chao, and Shannon are fungal alpha diversity indices; *Kazachstania, Aspergillus, Scopulariopsis*, and *Wallemia* are key fungal genera; open field center time and open field center enter are indicators in the open field test; EPM open arm enter, EPM OE%, EPM open arm time, and EPM OT% are indicators in the elevated plus maze test.

In the current investigation, Spearman correlation analysis was utilized to create correlation heatmaps (refer to Figure 11) that depict the interrelationships among gut fungal alpha diversity indices, essential fungal genera, and indicators of anxiety-like behaviors in mice. This methodology seeks to clarify possible connections between the fungal community and behavioral traits. The false discovery rate (FDR) correction technique was implemented during this correlation analysis. In the generated heatmap, the color red signifies a positive correlation, whereas blue represents a negative correlation; the degree of color intensity correlates with the absolute value of the correlation coefficient. Statistical significance is indicated by asterisks (^*^P < 0.05, ^**^P < 0.01, and ^***^P < 0.001). The rows and columns of the heatmap represent diversity indices/fungal genera and behavioral indicators, respectively, with the diagonal reflecting the perfect positive correlation of each variable with itself (correlation coefficient = 1) (Figure 12).

#### 3.3.2 Result analysis

##### 3.3.2.1 Association between fungal alpha diversity index and behavioral phenotype

Fungal alpha diversity metrics, including Simpson, Chao, and Shannon, showed substantial correlations with one another (*r* > 0.8, *P* < 0.01 or *P* < 0.001), indicating a strong level of internal consistency across these metrics. The Chao metric is utilized as an indicator of species richness present within the gut mycobiota. Conversely, the Shannon and Simpson metrics reflect microbial diversity, where a higher Shannon score and a lower Simpson score signify greater diversity. Importantly, the Chao and Shannon metrics were positively associated with specific anxiety-related behavioral indicators noted in the open field test and the elevated plus maze (EPM; *P* < 0.05). These results suggest that a greater overall abundance and diversity of gut fungi correlate with diminished anxiety-like behaviors in mice.

##### 3.3.2.2 Association of key fungal genera with fungal alpha diversity indices

*Kazachstania* displayed a notable negative association with the Chao index (*r* = −0.76, *P* < 0.01) and also showed a negative relationship with the Shannon index (*r* = −0.69, *P* < 0.05). On the other hand, *Aspergillus* revealed an exceptionally strong positive association with both the Chao and Shannon indices (*P* < 0.001), while it exhibited a significant negative correlation with the Simpson index (*r* = −0.79, *P* < 0.01). Furthermore, *Scopulariopsis* demonstrated a significant positive correlation with the Chao index (*r* = 0.83, *P* < 0.01) and maintained a positive relationship with the Shannon index (*r* = 0.72, *P* < 0.05). *Wallemia* was positively correlated with both the Chao and Shannon indices (*P* < 0.05) but showed a negative correlation with the Simpson index (*P* < 0.05). The analysis revealed that greater abundances of *Aspergillus, Scopulariopsis*, and *Wallemia* were linked to increased overall alpha diversity of fungi, while a higher concentration of *Kazachstania* was associated with lower overall fungal alpha diversity. This implies that *Aspergillus, Scopulariopsis*, and *Wallemia* might contribute to the preservation of diversity within the gut fungal community.

##### 3.3.2.3 Association of key fungal genera with behavioral phenotypes

*Kazachstania* demonstrated an inverse relationship with the duration spent in the center of the open field (*P* < 0.05) and showed some non-significant trends in correlation with different behavioral metrics. In contrast, *Aspergillus* revealed a positive association with several behavioral measures, including the frequency of entries into the center of the open field, duration spent in open arms on the EPM, and the percentage of time allocated to the open arms on the EPM (*P* < 0.05 or *P* < 0.01). Similarly, *Scopulariopsis* exhibited positive correlations with various EPM behavioral measures, such as the number of entries into the open arms, the percentage of time in the open arms, and the percentage of open entries (*P* < 0.05 or *P* < 0.01). On the other hand, *Wallemia* showed some non-significant correlational patterns with a range of behavioral indicators. The growth of *Kazachstani*a might be associated with behaviors indicative of increased anxiety. Furthermore, the presence of *Aspergillus, Scopulariopsis*, and *Wallemia* could potentially elevate the activity levels of mice during behavioral assessments and reduce anxiety-related traits.

#### 3.3.3 Conclusion

The variety within the gut fungal community, evaluated through the Chao and Shannon indices, has been noted to decline, coinciding with a decrease in the populations of *Aspergillus, Scopulariopsis*, and *Wallemia*, which is linked to increased anxiety-related behaviors in mice. In contrast, a higher presence of *Kazachstania* appears to correlate with reduced exploratory actions. These results indicate that states of anxiety could interfere with the gut mycobiome, leading to anxiety-like behaviors. Nevertheless, the direct connection between disturbances in the gut mycobiome and anxiety-like behaviors is still unclear and requires more thorough investigation. Furthermore, *Aspergillus, Scopulariopsis* and *Wallemia* could be critical candidate fungal genera, providing essential information for future mechanistic studies.

### 3.4 Co-occurrence network analysis

**Figure 13:**
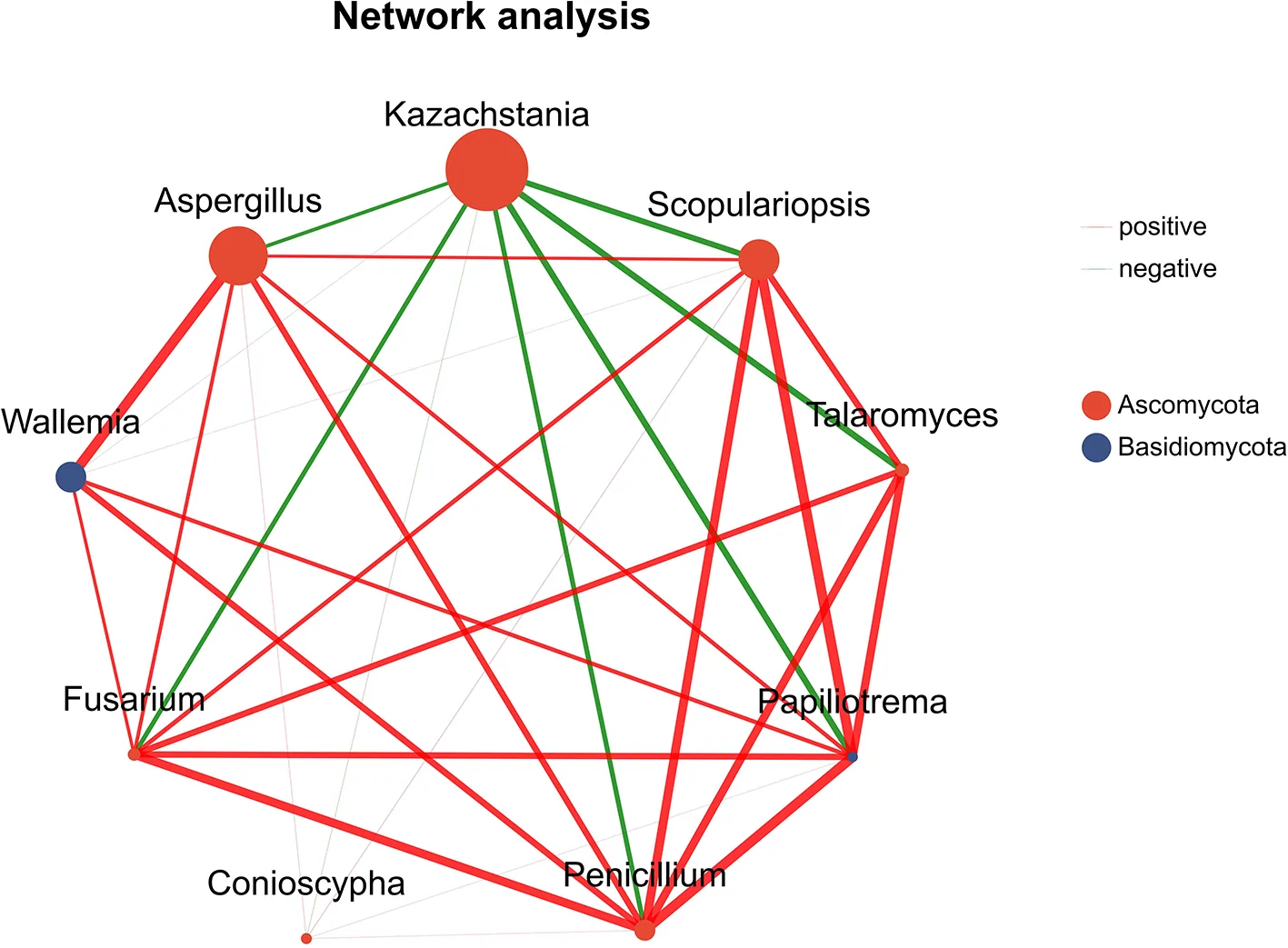
The co-occurrence network diagram showing mouse intestinal fungi at the genus level. The points in this illustration signify the predominant fungal genera identified within the fecal fungal community. The colors of the nodes are classified according to the fungal phylum, with red nodes representing *Ascomycota* and blue nodes indicating *Basidiomycota*. The legend clarifies that red lines represent significant positive correlations (co-occurrences) between two fungal genera, whereas green lines reflect significant negative correlations (competitive mutual exclusion) among pairs of fungal genera. This network includes a total of seven genera from *Ascomycota* (*Kazachstania, Aspergillus, Scopulariopsis, Fusarium, Conioscypha, Talaromyces*, and *Penicillium*) and two genera from *Basidiomycota* (*Wallemia* and *Papiliotrema*). *Kazachstania* serves as the central hub within the network, linking to all other fungal genera. Among these links, the relationships involving *Kazachstania* and the other genera, such as *Aspergillus, Scopulariopsis, Fusarium, Conioscypha, Talaromyces, Penicillium, Wallemia*, and *Papiliotrema*, are characterized by green negative-correlation lines. In contrast, all connections between any two other fungal genera, aside from *Kazachstania*, are illustrated as red positive-correlation lines.

To investigate the natural ecological interactions between dominant genera of gut fungi, we developed a co-occurrence network at the genus level, grounded in significant correlation coefficients. The nodes within the network were classified into two primary fungal phyla: red nodes identified *Ascomycota*, comprising *Kazachstania, Aspergillus, Scopulariopsis, Fusarium, Conioscypha, Talaromyces*, and *Penicillium*, while blue nodes represented *Wallemi*a and *Papiliotrema*, which are classified under *Basidiomycota*. The colors of the lines indicate the direction of the correlations: solid red lines represent significant positive co-occurrence associations, while solid green lines indicate significant negative ecological competition or mutual exclusion associations. Through topological analysis, it was determined that *Kazachstania* served as the central hub taxon, displaying the highest connectivity within the network, with green negative correlation lines connecting it to all other fungal genera depicted (Figure 13). This indicates that *Kazachstania* likely antagonizes all fungi present in the community through competitive ecological niches. Conversely, among the fungal taxa of *Aspergillus, Scopulariopsis, Fusarium, Conioscypha, Talaromyces, Penicillium, Wallemia*, and *Papiliotrema*—excluding Kazachstania—all pairwise interactions exhibited positive red co-occurrence associations, thereby establishing a synergistic and stable fungal subcommunity.

The characteristics of the network are in close alignment with the findings related to the differential mycobiota from this research, suggesting that chronic unpredictable stress significantly reduces the abundance of commensal fungi, including *Aspergillus, Scopulariopsis*, and *Wallemia*. This reduction directly disrupts the equilibrium among fungi needed for their synergistic coexistence. At the same time, the wide-ranging competitive inhibitory capabilities of fungi against *Kazachstania* are significantly diminished, creating a substantial amount of ecological space within the intestines. Consequently, this situation enables *Kazachstania* to grow extensively and without restriction, establishing itself as the predominant fungal species in the gut.

## 4 Discussion

### 4.1 Statistical significance in a CUMS pathogenic environment

To improve the specificity and practicality of biomarkers for future research, taxa at each branch of the sequencing were selected for LEfSe analysis, with an LDA score exceeding 5. Noticeable differences were observed in the characteristic fungal taxa between the Con group and the CUMS group. In the Con group, the most abundant signature fungi primarily included *Aspergillus* (which had the highest LDA score surpassing 5), *Scopulariopsis*, and *Wallemia*. In contrast, the CUMS group exhibited a more limited diversity, predominantly featuring *Kazachstania* (recording an LDA score of 5.5), which stood out as the most significant differential taxon within this group. Subsequent analysis, alongside genus-level differential assessment using Welch's *t*-test (refer to Figure 10), demonstrated that *Kazachstania* had the most pronounced inter-group difference in abundance (*P* < 0.001), whereas most of the inhibited fungal genera (such as *Aspergillus, Scopulariopsis*, and *Wallemia*) showed lower significance levels (ranging from *P* < 0.05 to *P* < 0.01), with none achieving a difference as remarkably significant as *Kazachstania*. This statistical imbalance indicates that changes associated with CUMS in the gut microenvironment might apply selective pressures on the fungal community rather than causing random disruptions. Furthermore, while there is no direct evidence supporting specific biological interactions among *Aspergillus, Scopulariopsis*, and *Wallemia*, past studies of environmental microbiomes have indicated that these three species are saprophytic fungi sharing highly overlapping ecological roles (Dai et al., 2020). Given the concurrent decrease in abundance of these three genera noted in this study, it is proposed that chronic stress-induced changes in the gut environment might adversely affect the stability of such niche-similar microbial communities, thus inadvertently promoting the relative enrichment of *Kazachstania* from an ecological perspective.

### 4.2 From diverse symbiosis to single-species dominance

Under CUMS induction, mice show a marked imbalance in their gut fungal community, with most species being suppressed and a single dominant species emerging. In contrast, the Con group exhibits a diverse multi-symbiotic pattern within their gut fungal community. Analyzing species-level abundance at the genus tier (Figure 9) indicates that various fungal genera, such as *Aspergillus, Scopulariopsis, Wallemia*, and *Talaromyces*, play a role in maintaining a relatively balanced ecological structure. Additionally, LEfSe analysis supports that the key biomarkers for the Con group encompass *Aspergillus* (from *Eurotiomycetes*), *Scopulariopsis* (from *Sordariomycetes*), and *Wallemia* (from *Basidiomycota*; Figure 11). This highlights that a healthy gut fungal community showcases diversity at higher taxonomic levels. Conversely, in the CUMS group, this diverse structure experiences a significant alteration. Genus-level differential analysis indicates a heightened prevalence of *Kazachstania* alongside reduced levels of *Scopulariopsis, Aspergillus*, and *Wallemia*. This observation is corroborated by LEfSe analysis, which reveals that the CUMS group is dominated by *Kazachstania*, while the Con group reflects a distribution across various classes, orders, and families.

Correlation analysis further indicated that lower abundances of *Scopulariopsis* and *Aspergillus* displayed positive correlations with the Chao index, Shannon index, and various elevated plus maze behavioral measures (*P* < 0.05 or *P* < 0.01). This suggests that in conditions of chronic unpredictable mild stress (CUMS), there is a significant structural alteration in the intestinal ecological landscape of mice, which is marked by the reduction of most microbial taxa alongside the prominent prevalence of *Kazachstania*. This noteworthy imbalance signifies a typical state of severe dysbiosis within microbial ecology (Krishnapriya et al., 2025), paralleling the condition of small intestinal bacterial overgrowth within the gut bacterial community; however, such a distinctly dominant pattern is comparatively rare in the context of fungal studies.

### 4.3 Speculative analysis of potential ecological associations among core fungal genera

The shift from a “multivariate symbiosis” approach to one centered on a “single dominant” model suggests that changes in the gut environment triggered by chronic unpredictable mild stress (CUMS) do not uniformly affect all fungal taxa. Rather, they display a specific bias that varies by taxon. This bias may involve a nuanced alignment between the ecological niche characteristics of different genera and the environmental changes induced by stress. The structural attributes of the fungal co-occurrence network provide a thorough ecological interpretation of how CUMS stress reorganizes fungal communities and sheds light on the relationships between mycobiota and behavioral metrics. During physiological equilibrium, fungi such as *Aspergillus, Scopulariopsis*, and *Wallemia* form extensive positive co-occurrence networks that collaboratively sustain a high level of community diversity, with their presence showing a positive correlation to alpha diversity. Additionally, greater populations of *Aspergillus* and *Scopulariopsis* have been associated with a decrease in anxiety-like behaviors in mice. Within the community, two types of interactions are evident: positive facilitation between fungi like *Aspergillus, Scopulariopsis*, and *Wallemia*, while *Kazachstania* competes globally with other fungi for niche resources. Following the CUMS modeling, populations of *Aspergillus, Scopulariopsis*, and *Wallemia* significantly decreased, which disrupted the synergistic network and diminished overall fungal diversity. This reduction also lessened the inhibitory effects on *Kazachstania*, leading to its marked increase; as a result, the harmonious symbiotic network collapsed, resulting in a dysbiotic community dominated by *Kazachstania*. The subsequent analysis speculates on the potential ecological relationships among the four genera: *Kazachstania, Aspergillus, Scopulariopsis*, and *Wallemia*, grounded in the established ecological and functional traits of each genus.

Some varieties within the *Kazachstania* telluris species complex are classified as typical fungal pathobionts (Iliev et al., 2024; Liao et al., 2024). The term “pathobiont” refers to resident commensal microorganisms that inhabit the host gut; these microorganisms stay non-pathogenic when the host's physiological homeostasis is intact and only show pathogenic potential when the internal conditions of the host are altered, such as in cases of chronic inflammation or compromised immune function. Their relationship with the host is characterized by a dynamic spectrum and cannot be easily classified as either “commensal” or “pathogenic” (Chow et al., 2011).

While in a state of physiological balance, *Kazachstania* functions as a commensal fungus that ordinarily resides in the gastrointestinal tract of experimental mice and can be passed from mother to offspring. The fungus's proliferation is constrained by both competition from other microbes in the gut and the functioning mucosal immune surveillance system, which maintains a low level of colonization. Under these conditions, *Kazachstania* has only minimal immunomodulatory effects, does not penetrate intestinal epithelial cells, and does not cause mucosal inflammatory damage (Iliev et al., 2024; Sekeresova Kralova et al., 2024).

Chronic unpredictable mild stress (CUMS) modeling leads to several homeostatic disturbances in mice, which include pathological alterations such as immune dysfunction, impairment of intestinal barrier integrity, and ongoing low-grade chronic inflammation (Wu et al., 2021; Wei et al., 2019). In the setting of internal environmental imbalance, the gut-resident *Kazachstania* demonstrates significant growth in the intestine, while the population of other fungal taxa decreases concurrently, resulting in *Kazachstania* becoming the predominant gut fungus. This finding closely aligns with our research (Kurtzman et al., 2005; Zhang et al., 2022b; Hiengrach et al., 2023).

Our correlation analysis revealed that the abundance of *Kazachstania* showed only marginal trends in correlation with certain anxiety-like behavioral indicators in mice, with no substantial correlation identified between them. Thus, we propose that the homeostatic imbalance induced by CUMS may promote the pathological overgrowth of *Kazachstania*; however, the significant proliferation of this fungus does not appear to directly worsen anxiety-like behavioral traits in mice.

The colonization of the gut by *Aspergillus* and *Scopulariopsis* might provide potential protective roles. In sharp contrast to the singular dominant genus *Kazachstania*, significant suppression of both *Aspergillus* and *Scopulariopsis* was observed in the CUMS group. Research has indicated that *Aspergillus* is a vital element of the human gut fungal community and acts as a stable colonizer in the intestines of healthy individuals (Hallen-Adams and Suhr, 2016). In studies involving non-human primates, *Aspergillus* has also been recognized as a prominent fungal genus found in higher concentrations in the colon (Yang et al., 2023). Functionally, *Aspergillus* cristatum has demonstrated substantial protective effects in models of colitis by safeguarding the intestinal barrier, interfering with the NLRP3 signaling pathway, and lowering levels of the pro-inflammatory cytokines IL-6 and TNF-α (Wang et al., 2025). Moreover, *Scopulariopsis* has been noted as one of the leading genera in the chicken gut fungal community, making up a significant portion of the gut mycobiota along with *Aspergillus*. Furthermore, *Scopulariopsis* sp. isolated from the gastrointestinal tract of fish is known to produce scopularide-class lipopeptides (Elbanna et al., 2019), indicating that this genus might contribute to the maintenance of intestinal microecology through its secondary metabolites. In conclusion, both *Aspergillus* and *Scopulariopsis* may have important functions in sustaining barrier integrity and microecological balance in a healthy gut, potentially reducing anxiety-like behaviors. Their diminished presence could signify the selective suppression of beneficial fungal taxa by the intestinal conditions associated with CUMS.

*Wallemia* was recognized as a distinct biomarker taxon in the control group through LEfSe analysis. In non-human primates, it has been noted that *Wallemia*, in conjunction with *Aspergillus*, is a fungal genus that is prevalent in the colon (Yang et al., 2023). Additionally, in a model of DSS-induced colitis, *Wallemia's* abundance was significantly diminished (Qiu et al., 2015), which corresponds with the observed declining trend of *Wallemia* in the CUMS group within this research. This implies that *Wallemia* might respond sensitively to conditions of intestinal inflammation or stress. Some research indicates that Wallemia could serve a potential function in modulating both beneficial and harmful bacteria (Arribas-Rodríguez et al., 2025). In the context of CUMS modeling, the relative abundance of *Wallemia* saw a decrease, potentially worsening the irregular growth of *Kazachstania*.

In conclusion, the healthy gut mycobiota includes relatively stable residents such as *Aspergillus, Scopulariopsis*, and *Wallemia*. The concurrent suppression of these genera in the chronic unpredictable mild stress (CUMS) group suggests that they may occupy an ecological niche that is particularly vulnerable to changes in environmental conditions. Alterations in the gut microenvironment linked to CUMS can lead to synchronous suppression of these genera, which occupy comparable ecological spaces. *Kazachstania* benefits from an inherent advantage in colonization, being capable of thriving without bacteria and resisting colonization by other fungal species, which may allow it to quickly fill the ecological void left by a decrease in other microorganisms. Moreover, since the genus *Kazachstania* has demonstrated significant competitive abilities against other fungi, it has the potential to further limit the presence of other fungal genera during its expansion by producing metabolites or occupying available niches, thereby enhancing this cycle.

### 4.4 Analysis of study limitations

This research, utilizing the Chronic Unpredictable Mild Stress (CUMS) model in mice, provides preliminary evidence indicating that chronic stress may lead to dysbiosis in the gut mycobiome, which correlates with anxiety-like behaviors observed in the subjects. Nonetheless, it is important to note several limitations that warrant careful interpretation of the findings.

To begin with, the experimental model and sample design reveal shortcomings. The study focused solely on male C57BL/6 mice, which overlooks potential gender-related differences in responses to stress and fungal colonization in the gut. Moreover, the design included only a control group and a CUMS group, omitting a gradient of stress duration or intervention/rescue groups. While the 3-week CUMS modeling is valid, it mostly reflects mild to moderate depressive changes and does not replicate the ongoing pathological effects associated with long-term severe depression. Regarding the samples, colonic contents were obtained only at a single terminal time point following the establishment of the model, without any longitudinal time-series data. This limitation impedes the assessment of the dynamic relationship between fungal dysbiosis and anxiety-like behavior over time. Additionally, mucosa-adherent fungi were neither isolated nor analyzed, leading to a lack of data concerning mucosal fungi that play a crucial role in modulating intestinal immunity. Additionally, the investigation was limited to a single set of animal experiments, lacking an independent validation cohort or human clinical samples, which constrains the applicability of the findings in clinical settings.

Moreover, the system used for microbiome detection and statistical analysis is inadequate. The study focused solely on assessing the relative abundance of fungi, which prevents a clear determination of whether an increase in the abundance of specific fungi indicates true proliferation or simply a proportional alteration due to a decrease in other fungal species, as it lacks empirical evidence from absolute quantification and strain culturing. Furthermore, the ITS sequencing process did not include blank controls or measures for filtering contamination, making the outcomes susceptible to influences from environmental microbial contamination. In addition, intestinal bacterial communities were not analyzed simultaneously, leaving it unclear whether the changes in gut fungi were a direct result of chronic stress or merely a secondary effect of bacterial dysbiosis.

Finally, the broader application of the study's findings is restricted. This experiment utilized specific pathogen-free (SPF) mice with well-defined genetic backgrounds and controlled living conditions, whose gut fungal composition and stress response patterns differ considerably from the intricate physiological traits and living conditions of humans. As a result, the conclusions derived from mouse experiments are not directly applicable to clinical populations facing anxiety and depression.

### 4.5 Prospects for future research

Short-term objectives involve performing functional experiments to unravel causal mechanisms. This includes the application of mucosal fungal sequencing to examine the variations in fungal communities between the lumen and mucosa, along with the use of fungal qPCR for absolute quantification to address the shortcomings of relative abundance analysis. Through rescue experiments—such as mono-colonization of germ-free mice, fecal mycobiota transplantation, and antifungal treatments—we intend to elucidate the roles of *Kazachstania, Aspergillus*, and *Scopulariopsis* in modulating anxiety-like behavior. Additionally, we plan to incorporate 16S sequencing to explore bacterial-fungal cross-kingdom interactions and evaluate intestinal barrier integrity, immune pathways, and fungal metabolomics. This approach will enable us to construct a regulatory framework represented as “chronic stress—dysbiosis of gut mycobiota—decreased protective fungi—anxiety-like behavior.” Simultaneously, we will isolate and culture fecal fungi, particularly the type *Kazachstania*, and utilize *in vitro* co-culture methodologies to investigate the interspecific competition mechanisms that allow it to suppress the growth of *Aspergillus, Scopulariopsis*, and *Wallemia*.

For the long-term improvement of animal models and enhancement of clinical applicability, our plan includes the addition of female mouse subgroups, varying durations of stress, and groups for stress recovery. Furthermore, we will carry out mycobiome sequencing in population cohorts to corroborate findings from animal studies and evaluate the feasibility of gut fungi as non-invasive biomarkers for mood disorders. By focusing on key functional strains, our goal is to devise microecological intervention formulations targeting gut fungi, thereby offering novel strategies for both the prevention and treatment of mood disorders associated with chronic stress.

## 5 Conclusion

This research illustrates for the first time that chronic unpredictable mild stress (CUMS) can trigger a significant ecological shift in the gut fungal population, notably marked by the prevalence of *Kazachstania* and the extensive suppression of other fungal genera. By corroborating the findings of this research with pre-existing studies, we illustrate that stress induced by CUMS may activate the dominance potential of *Kazachstania*, classifying it as an opportunistic dominant fungus that is dependent on environmental conditions. Moreover, we present the hypothesis that the homeostatic disruptions caused by CUMS can lead to the pathological overgrowth of *Kazachstania*, although this does not directly worsen anxiety-like behavioral traits in mice. Additionally, we explore the idea that *Aspergillus, Scopulariopsis*, and *Wallemia* play roles in sustaining intestinal homeostasis and in counteracting anxiety-like behaviors. This observation contests the earlier conceptual model, which ascribed alterations in the fungal community solely to a decrease in diversity, suggesting instead that the dominance of single species within the gut fungal community might be more reflective of pathological conditions than merely reduced diversity. It opens up a new theoretical framework and pathways for future experiments focused on how CUMS influences host health through modifications in gut microecology.

## Data Availability

The datasets presented in this study can be found in online repositories. The names of the repository/repositories and accession number(s) can be found in the article/Supplementary material.
